# Environmental Risk Perception, Risk Culture, and Pro-Environmental Behavior

**DOI:** 10.3390/ijerph17051750

**Published:** 2020-03-07

**Authors:** Jingjing Zeng, Meiquan Jiang, Meng Yuan

**Affiliations:** 1 Zhongnan University of Economics and Law, Wuhan 430073, China; jjzeng@zuel.edu.cn (J.Z.); jiangmeiquan3@gmail.com (M.J.); 2 Department of Political Science, Northern Illinois University, DeKalb, IL 60115, USA

**Keywords:** environmental risk perception, cultural theory of risk, pro-environmental behavior

## Abstract

Mixed evidence exists regarding the relationship between environmental risk perception and pro-environmental behavior. This study uses an existing online survey conducted by the Center of Ecological Civilization (CEC) of China University of Geosciences from December 2015 to March 2016 and examines how cultural bias influences environmental risk perception and behavior. We found that an individual’s pro-environmental behavior is not only influenced by environmental risk perception, but also by his or her cultural worldviews. Built on culture theory (CT), our empirical results suggest that young Chinese people are more located in “high-group” culture, where egalitarian culture and hierarchical culture dominate. The higher scores of hierarchical and egalitarian cultures of Chinese youth, the more likely they are to protect the environment. Moreover, the relationship between cultural worldviews and pro-environmental behaviors are mediated by perceived environmental risks.

## 1. Introduction

Environmental problems have become a serious social risk that need to be urgently solved all over the world. The public’s voluntary pro-environmental behavior, including that of youths, is critical to achieve environmental sustainability [1,2,3,4,5,6]. Moreover, because college students will be the leaders of society and decision makers of public institutions, understanding their environmental concerns and behavior has great value for policymakers who want to improve environmental policy compliance and governance [7].

Environmental risk perception stimulates people’s sense of urgency and responsibility to protect the environment and encourages more environmental protection behaviors [1,2,8]. However, other scholars have found that there is a discrepancy between environmental risk perception and environmental protection behaviors [9,10]. Individuals who possess the same level of perceived environmental risk can have inconsistent behaviors in environmental protection [11,12]. In addition, existing research mainly built on the rational choice model (RCM) or relied on individual cost-benefit analysis [12,13]. Like other cultural scholars, this paper tries to look to what RCM cannot explain by using the culture theory [14,15,16].

According to culture theory (CT), people have different preferences of how the society should be organized, which then affects how they evaluate and respond to risk [17]. Existing studies have confirmed the relationship between cultural worldviews and risk perception suggested by CT [18,19,20,21]. Other scholars further found that risk perception, as a mediator, can influence the relationship between culture and pro-environmental behavior [11,22]. However, existing studies did not answer whether culture can influence pro-environmental behavior directly. This study fills this gap by investigating how culture influences pro-environmental behavior directly. This study also tries to test the generalizability of CT by applying CT to non-Western countries.

This paper uses an existing online survey conducted by the Center for Ecological Civilization of China University of Geosciences (CEC) from December 2015 to March 2016. Our sample includes 8084 students from 152 universities in 30 provinces across China, who were surveyed on the Internet. This research makes contributions in four aspects. First, this large-scale data allows us to map the distribution of environmental risk perception and pro-environmental behavior among young people in China. Second, this paper is the first research testing the influence of individuals’ cultural worldviews on pro-environmental behavior in China. More specifically, we test the effects of culture worldviews, environmental risk perception and other control variables, including knowledge and demographics, on pro-environmental behavior. Third, this paper analyzes how the relationship between risk culture and pro-environmental behavior is mediated by an individual’s cultural bias. Fourth, Douglas (1982) used the “grid-group” framework to analyze how the relations between individuals and society determine people’s perception of risk. CT was then introduced by Wildavsky to study politics in the United States and was applied to many western countries [23]. However, there is only a few pieces of research on China [21,22]. This study will contribute to improve CT’s generalizability by applying CT to Chinese context and providing some implications for measuring culture based on existing research.

The paper starts with a review of CT and its application in China. Based on the theory and existing research on the relationships among cultures, environmental risk perception, and pro-environmental behavior, we suggest three sets of research hypotheses in Section 2. Section 3 introduces data, variables, and methods used to test our hypotheses. Section 4 describes the result of our data testing and examines its robustness. Section 5 is the conclusion of the paper.

## 2. Theoretical Background and Hypotheses

### 2.1. Cultural Theory of Risk and Differences of Chinese Youth Culture Types

**Figure 1:**
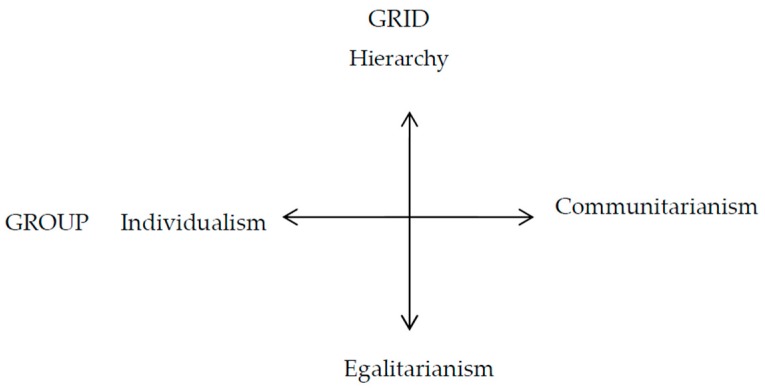
Douglas’ (1970) “grid-group” social type quadrant.

**Figure 2:**
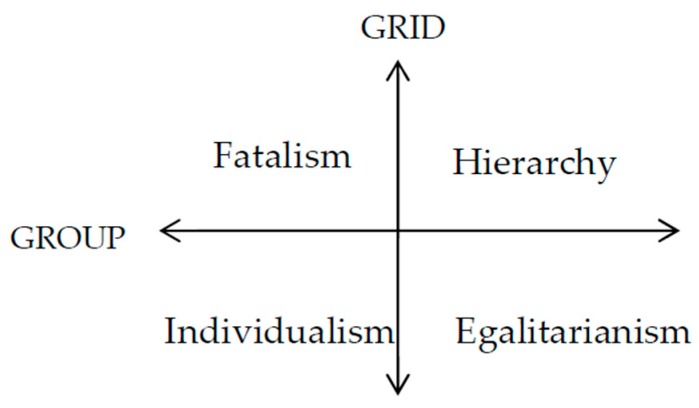
Dake’s (1992) “grid-group” risk culture type quadrant.

“Grid-group” cultural theory of risk (Figure 1) was proposed by Douglas (1970), a religious anthropologist. Douglas’s (1970) “grid-group” framework helps us to understand how individuals’ perception and behavior are influenced by four different social types with different core values. Influenced by “grid-group” framework, subsequent researchers further revised and clarified four types of culture. For example, Dake (1992) improved Douglas’s (1970) model and combined the dimensions of grid and group to develop four types of risk culture (Figure 2): fatalism, hierarchy, individualism, and egalitarianism.

In culture theory, “grid” dimension refers to rules that connect an individual with another on an ego-centered basis [24]. Grid is the preference of the social structures that rely on role- or class-based rules (high grid) versus those emphasizing equal opportunity and status among individuals (low grid) [22,25]. “Group” dimension refers to the extent that one person is bound by a group. Group is reflected in the preference of social structures that encourage cooperation, collectivism, and collective interests (high group) versus those that support competition and personal interests (low group) [24,25]. However, unlike other psychometric scales, cultural theory suggests that grid and group dimensions vary separately and independently on their own [26,27,28].

In Chinese social and cultural systems, the collective thinking makes Chinese people emphasize collective interests over personal interests and connect with each other closely. In addition, China is a country in which Han ethnicity (Han Zu) accounts for a majority of the overall Chinese population. Studies have shown that countries with a single ethnicity tend to prefer collectivism culture [25]. In the political context, China’s one-party system and centralized government can increase people’s preference for bureaucracy and social system that emphasizes role or class stratification. In fact, not much literature has analyzed and compared the cultural characteristics of different countries in terms of grid and group dimension. As an exception, Chai et al. used the World Values Survey and found that the culture of China is placed in the high group and high grid quadrant: hierarchy [29].

Our research population is Chinese youth with college education or above. Some existing research found that the Chinese younger generation tends to be more individualistic than the older generation [30]. Others have suggested that younger generations are more likely to participate in or support social environmental movements that are consistent with egalitarian values [25]. Therefore, we expect that Chinese young people are more likely to be located in the high-group quadrant.

**Hypothesis** **1.** *Compared with other culture types, the culture of Chinese youth is more likely to be Hierarchy and Egalitarianism.*

### 2.2. Cultural Worldviews and Pro-Environmental Behavior

According to the CT, people have different preferences of how the society should be organized, which influences how they evaluate and respond to risk [17]. Different from the traditional rational model which assumes that people rationalize their choices based upon cost benefit analysis, culture theory assumes that people think and act in certain ways that are consistent with their culture [15,16,25,31]. In other words, individuals will view the issue that opposes their preferred lifestyles as dangerous, and act on their perceived danger from the issue [28,32]. Values applied by each of the four cultures are constraining decisions and behaviors and are difficult to change [33]. Therefore, we will analyze four different cultural types separately to infer their guiding roles in behavior.

Because individuals who prefer an egalitarian culture believe that everyone in society is equal [13,14,15], they are more likely to accept ideas supporting equal and fair treatment between human and nature [34,35] and are more willing to seek common interests for both the development of human society and the natural environment. Therefore, they tend to worry about environmental risks and be more active in environmental protection [36,37,38,39,40]. In contrast, individuals who prefer an individualistic culture are more concerned about freedom than equality. They are more tolerant of environmental risks [41]. Because they believe that regulations protecting the environment will simultaneously have restrictions on the development of business and industry, they view environmental protection as threats to freedom, and this view will further decrease their pro-environmental behavior [41]. Individuals who prefer a hierarchical culture believe that everyone in society has their own role and status [42], and environmental risk is seen as a “hidden symbolic cultural model of social elite status and authority” [29]. They trust authority and believe that over-worrying about environmental risks is challenging the status quo and authorities. Therefore, they are more likely to devalue environmental protection, perceive environmental risk as low, and less likely to demonstrate pro-environmental behavior. However, some research found that hierarchists can closely align with the egalitarians in some environmental risk perceptions such as climate change [37]. Since the hierarchists respect experts and authority, they are more likely to accept information from environmental experts or government authorities [32]. As the Chinese government has been increasing environmental risk propaganda in recent years, it may send signals to hierarchists that environmental protection is important and they should take actions to protect the environment. Fatalism is a culture that favors unpredictable actions. Because fatalists are unlikely to maintain a stable position in life, it has been proven that they have little to do with environmental problems [8,43,44]. Consequently, fatalism is often excluded from many studies that predict environmental protection behaviors.

Any individual can be viewed as a hybrid of the four types of culture [25], and an individual’s environmental perception and behavior are influenced by the mixture of different cultural types [21]. Therefore, we created four indicators for each of the four types of respondents’ risk culture [45]. We expect:

**Hypothesis** **2a.** *Cultural worldviews are significantly associated with individual pro-environmental behavior. Hierarchical and egalitarian cultures will increase people’s pro-environmental behavior.*

**Hypothesis** **2b.** *Individualistic culture will decrease people’s pro-environmental behavior.*

**Hypothesis** **2c.** *Fatalist culture will not influence people’s pro-environmental behavior.*

### 2.3. Cultural Worldviews, Environmental Risk Perception, and Pro-Environmental Behavior

Environmental risk perception is an individual’s understanding of the importance and urgency of environmental protection and the relationship between people and the environment. Individuals’ propensities to protect the environment can be dependent on their perceived environmental risk. For example, they may decide to change their behavioral habits and lifestyles to protect the environment when they perceive a high level of environmental risks. Pro-environmental behavior is a human behavior that reflects a relatively consistent eco-friendly propensity to buy, use or dispose of a particular product [46]. Pro-environmental behavior also refers to some general environmental behavior such as taking the initiative to understand the relevant information of ecological civilization, participating in ecological civilization activities, turning off the lights when not in use, and paying attention to garbage classification [47].

In addition to the finding that risk perception can directly influence pro-environmental behaviors, previous studies have also shown that the influence of cultural worldviews on pro-environmental behavior can be mediated by environmental risk perception [8,11]. According to culture theory, specific behaviors result from specific attitudes that are constrained by culture [8,11]. Therefore, each of the four types of cultures will influence the levels of environmental risk perception, which will further influence the behavioral choices.

Prior studies on the relationship between cultural worldview and risk perception have found that individuals with fatalistic culture and individualistic culture rate risk lower than those with hierarchical and egalitarian culture [31,43]. In addition, research has found that environmental risk perception can explain the varieties of pro-environmental behavior [1,2,45,48,49], and individuals with higher awareness of environmental risk will demonstrate more pro-environmental behavior than those with lower awareness of environmental risk [50,51]. However, many scholars have found that there is a discrepancy between environmental risk perception and participation in environmental protection activities, which is referred to as attitude-behavior gap or value-action gap [9,10,11]. In this study, we expect:

**Hypothesis** **3a.** *Risk perception plays a mediating role in the relationship between risk culture and pro-environmental behavior.*

**Hypothesis** **3b.** *The higher the environmental risk perception of an individual is, the more pro-environmental behavior he or she has.*

## 3. Data, Variables and Methods

### 3.1. Data

Data source: our paper uses an existing online survey conducted by the Center of Ecological Civilization (CEC) of China University of Geosciences from December 2015 to March 2016. China University of Geosciences is a comprehensive university with diverse majors including earth system science, applied science and many interdisciplinary research areas. To collect a large-scale sample across different provinces, the Center for Ecological Civilization first set up an ecological survey team consisting of 50 students from different majors to ensure the diversity of respondents. Next, a “snowball sampling” approach was used. The students from the survey team recruited 250 college students from different provinces as the initial respondents. These 250 students were asked to answer the survey and send the survey to their middle school classmates who were studying in different regions or universities. This process was repeated multiple times until there were very few responses received. In order to ensure the randomness of sample distribution, each respondent was required to recruit no more than five participants, and these five participants should not have studied in the same province. The survey covered 152 universities in 30 provinces (excluding Tibet, Hong Kong, Macau, and Taiwan) and received a total of 14,097 responses. Among the participants in the survey, 66.14% are college students, 29.69% are graduate students and 4.5% are doctoral students. The survey was distributed by using the most popular social software in China: Tencent and Wechat.

Data screening: To ensure high quality of the data, the data screening of this paper includes three steps: first, we removed the observations with missing values; second, we removed the cases in which respondents answered the survey in less than three minutes; third, the illogical answers were removed. For example, those who chose government when they were asked “Who is responsible for the construction of ecological civilization?” but ranked government as lower than enterprises and individuals when they were asked to rank “Who should be responsible for construction of ecological civilization?” were removed. As a result, our final sample size included 8084 valid cases.

### 3.2. Methods and Variables

To investigate the determinants of pro-environmental behaviors of college students and the mediating effect of environmental risk on the relationship between culture and pro-environmental behavior, we constructed three multiple regression models:

(1) $$Behavior_{i}=\alpha_{0}+\alpha_{1}Culture_{i}+\overset{n}{\underset{k=1}{\sum }}\alpha_{2}Control_{i,k}+\mu_{1}$$

(2) $$Perception_{i}=\beta_{0}+\beta_{1}Culture_{i}+\overset{n}{\underset{k=1}{\sum }}\beta_{2}Control_{i,k}+\mu_{2}$$

(3) $$Behavior_{i}=\delta_{0}+\delta_{1}Culture_{i}\;+\;\delta_{2}Perception_{i}+\overset{n}{\underset{k=1}{\sum }}\delta_{3}Control_{i,k}+\mu_{3}$$

where ${\mathrm{Behaviror}}_{i}$ is the sum of scores of seven types of environmental actions. The respondents were asked to rate separately, using a 5-point scale, the level and frequency of pro-environmental behaviors. This research selected seven types of behavior from the survey, and these behaviors have also appeared in previous research on pro-environmental behaviors [46,47,49]. For example, we selected questions regarding respondents’ attention to the classification of the trash and environmental protection signs to measure purchasing and using of green product [46]. We also selected questions regarding turning off the lights when you leave the room and questions regarding their participation in environmental activities, including donations for eco-public activities and ecological civilization activities, to measure general pro-environmental behaviors [47,49]. Table 1 summarized our selected seven types of pro-environmental behavior (see Table 1). ${\mathrm{Perception}}_{i}$ denotes the perceived worries of environmental issues. Students were asked to answer whether they worry about the current overall ecological environment in China (5-point scale: 1: Definitely not; 2: Probably not; 3: Neutral; 4: Probably worry; 5: Definitely very worry).

${\mathrm{Culture}}_{i}$ is the individual’s risk culture type (including fatalism, hierarchy, individualism and egalitarianism). CT scholars have been debating whether individual cultural bias should be classified as a dominant culture in four quadrants or measured by four indices indicating different levels for each of the four types of culture. This paper argues that each individual should be treated as a hybrid of the four types of culture and uses four cultural indices separately to measure each of the cultures [16,17]. CT scholars have noticed that worldview is not an ideal measurement for culture and suggested that a combination of worldview and relational statement measures have better validity [52]. In this study, we select survey items that best approximated the four types of worldviews for two reasons (in Appendix A). First, existing research justified the value of selecting worldview survey items in existing research to measure cultures [29,53,54,55]. Second, research on Asian countries have found limitations and challenges by using measures developed for research in the United States [8,56]. Therefore, this research tries to select survey items that have better face validity. Questions regarding the role of authority and government and preference for self-interest over other values should have face validity [52]. In addition, while questions regarding attitude to economic activity are viewed as maybe having face validity in Swedlow et al.’s (2019) research, these questions have been used in some existing cultural research [53,57]. Therefore, we selected four survey questions to measure four types of culture: (1) What motivates you to protect the environment, (2) who should take the responsibility for environmental protection, (3) attitude toward commerce and industry, and (4) ranking of responsibility in environmental protection (Table 2) [14,21,28]. For each question, the respondent was assigned different scores for each question for each of the four types of cultures according to their answers (see more detail in Table 2). The scores for each question were then summed to create four cultural indices [45]. Because we use an existing research that was not designed to test culture, the respondents were not told explicitly that these questions will be used to measure their cultures. Therefore, we believe that response bias, such as social desirability bias, should be minimal.

X is the vector of controls, including knowledge, gender, grade, school, and the province in which the respondent attended her or his school. Age is not included in this questionnaire. However, this survey has a question of grade. Although grade and age are not the same thing, to some extent, grade should be correlated with age for university students in China. Consistent with previous cultural surveys in public opinion research [58,59], our study used two variables to measure knowledge. First, we used a master’s degree or doctorate as a proxy for “policy elite” or “intellectual.” Second, we created a dummy variable to indicate whether the respondents were majoring in an ecological environment related area.

The statistical analysis process of the findings is performed in the following way: first, we regressed pro-environmental behavior on culture to test the main effect of culture (Equation (1)). If $\alpha_{1}$ is significantly positive, Hypothesis 2 set for this study is valid and we can continue to carry out the next test. Second, we regressed pro-environmental behavior on environmental risk perception to check whether the $\beta_{1}$ is significant (Equation (2)). Third, we regressed pro-environmental behavior on both culture and environmental risk perception (Equation (3)). In this test, we will focus on the size and significance of $\delta_{1}$ in Equation (3). We compare the coefficient in different steps: if both $\beta_{1}$ and $\delta_{2}$ are significant and the value of $\delta_{1}$ becomes smaller, it means that risk perception plays a mediating role in which culture predict pro-environmental behavior (Hypothesis 3a). If $\delta_{2}$ is significantly positive, it means that Hypothesis 3b is valid, meaning that risk perception can increase the pro-environmental behavior.

## 4. Analysis and Results

### 4.1. Summary Statistics of Related Variables

**Figure 3:**
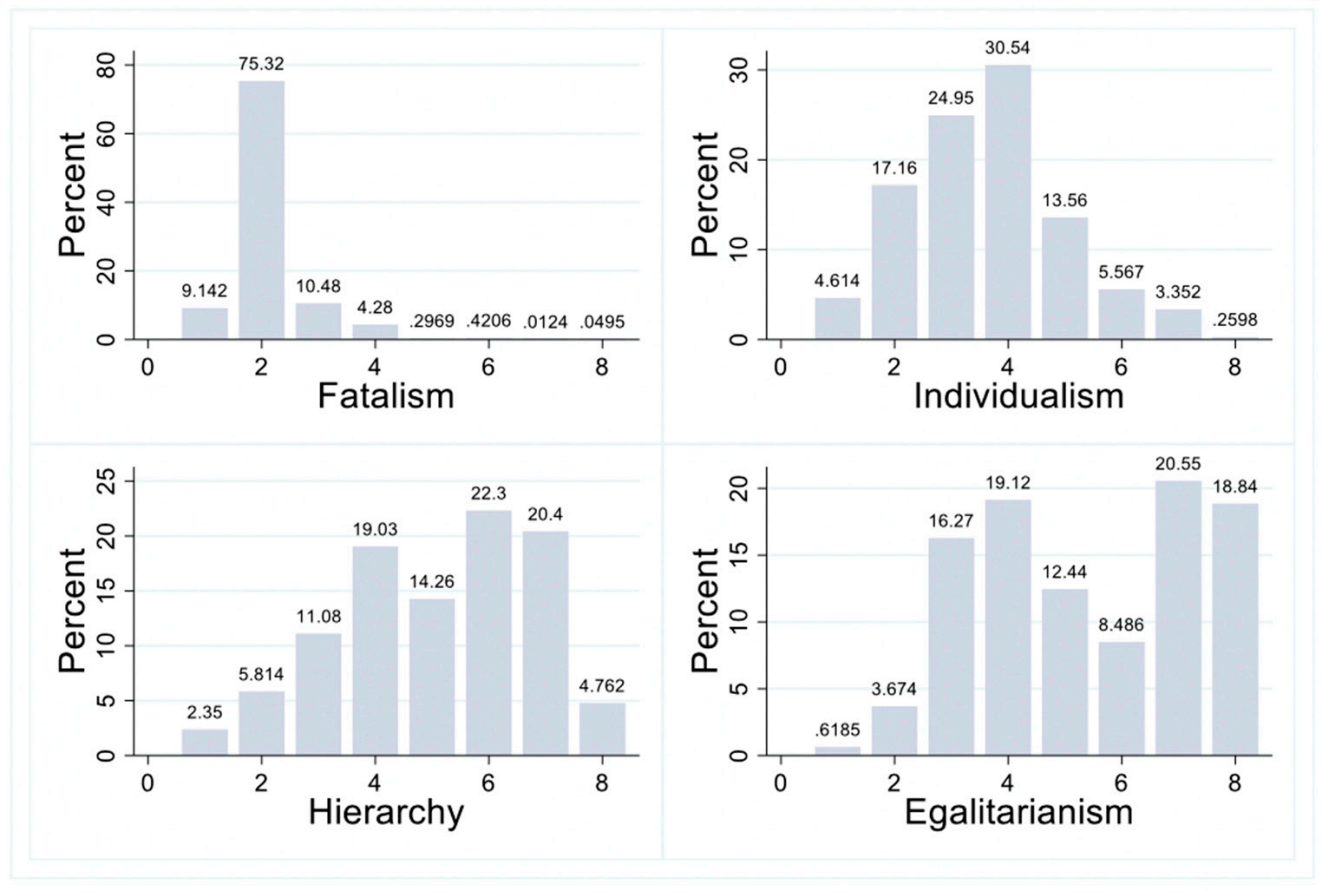
Distribution of Risk Cultural Biases (Average Frequency).

Table 3 reports the descriptive statistics of the variables, from which the overall characteristics of the sample data can be obtained: the score of pro-environmental behavior ranges from 7 to 28 and mean value is 22.995, which indicates that the mean value (average level) of seven types of pro-environmental behavior of college students is relatively high. However, the standard deviation is large, indicating that the differences in behavior among college students are large. In terms of the distribution of cultural bias, overall, Chinese youth has a highest mean value of egalitarian culture (5.409), followed by hierarchical culture (5.094), individualistic culture (3.627), and fatalism (2.128). This shows that Chinese youth are more likely to be hierarchists and egalitarians. Figure 3 further explains the distribution of different scores in four cultural bias among young Chinese people. X-axis is the score of individuals’ four cultural types and ranges from 1 to 8. Y-axis is the percentage of each score in different cultures. The overall distribution of hierarchical cultural scores are very similar with the egalitarian culture, and most of people have higher scores in egalitarian and hierarchical culture while most of people have lower scores in individualism and fatalism. There are 4876 people having a score of 5 or above for egalitarian culture, which accounted for 60.32% of respondents. Similarly, there are 7990 people having a score of 5 or more for hierarchical culture, which accounted for 61.73% of responses. This means that Chinese youth are more likely to be hierarchists and egalitarians.

**Figure 4:**
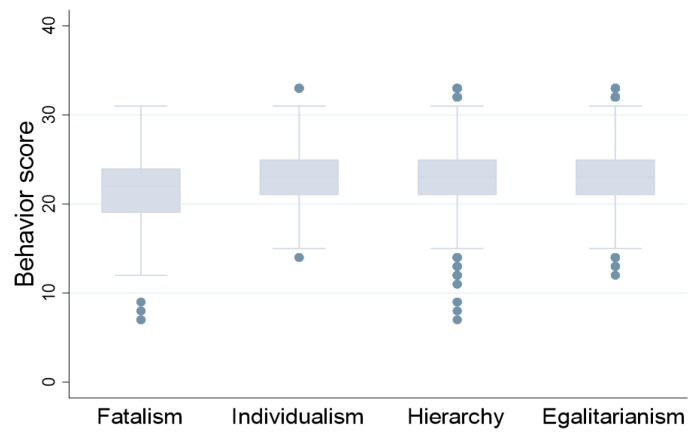
Risk Culture and Pro-environmental Behavior.

**Figure 5:**
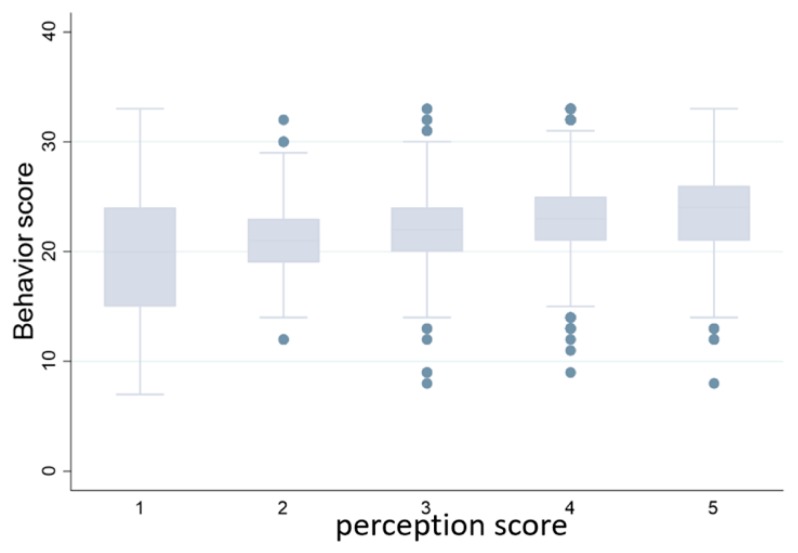
Risk perception and Pro-environmental Behavior.

Then, we used one-way ANOVA test to analyze whether there were differences in pro-environmental behaviors among four different cultures. The culture type was rated highest among the four cultures was coded as an individual’s dominant culture. Since the behavior distribution of individuals dominated by fatalistic culture did not follow the normal distribution (by analyzing its distribution), we only conducted variance analysis on the behavior among other three types of culture, and the results were as follows (Table 4):

The results suggest significant differences in pro-environmental behaviors among three cultural types. Figure 4 describes the score of pro-environmental behavior among four types of dominant cultures. Individuals who are affiliated with egalitarian culture and hierarchical culture have higher pro-environmental behavior score. In addition, Figure 5 shows the relationship between risk perception level and pro-environmental behavior. The level of pro-environmental behavior increases as environmental risk perception increases.

Table 5 reports correlations among our key variables. Most of correlation coefficients are between −0.5 and 0.5 (Although egalitarianism and hierarchy are negatively correlated at moderate level (−0.611), we would argue that it is reasonable given that hierarchy and egalitarianism share the attribute of high group.) It shows that there is no significant multicollinearity. In the preliminary analysis of the relationship between environmental risk perception and pro-environmental behavior, there is a positive correlation at the 1% level, which makes us more confident to test our hypothesis that there is a correlation between risk culture and pro-environmental behavior. At the same time, we can see the varieties of associations between four risk cultures and pro-environmental behaviors, with the egalitarian culture showing a significant positive correlation. However, it is worth noting that although fatalism has a significant negative correlation, it is not suitable for explanation due to the small sample size. 

### 4.2. Results

In this paper, multiple linear regression methods were used to explore the relationship between individual environmental risk perception and pro-environmental behavior. In order to ensure the validity of the regression results, we tested the assumptions of OLS regression, including linear correlation between independent variables and dependent variables (From the significance test of sample correlation coefficient, $r=\frac{\sum (X_{i}-\bar{X})(Y-\bar{Y})}{\sqrt{\sum {\left(X_{i}-\bar{X}\right)}^{2}}\sqrt{\sum {\left(Y_{i}-\bar{Y}\right)}^{2}}}$, *p* < 0.01) and the normally distributed error term (by testing the distribution of the residuals). We also used heteroscedasticity-robust standard error [60,61,62]. The regression results are shown in Table 6. The dependent variable in model 1 is risk perception, and model 1 was used to test the effect of culture worldviews on environmental risk perception. The dependent variable of Models 2–4 is pro-environmental behavior. Risk perception, culture and both of them were entered sequentially to investigate their impacts on pro-environmental behavior. More specifically, Model 2 tested the effect of environmental risk perception on pro-environmental behavior. Model 3 tested the effect of culture on pro-environmental behaviors. Model 4 tested the mediating effect of risk perception on the relationship between culture and pro-environmental behavior.

Hypothesis 2, that cultural worldviews are significantly associated with individual pro-environmental behaviors, was supported in Model 3. The results suggest that Chinese youth who have the higher scores of hierarchical and egalitarian are more likely to demonstrate pro-environmental behavior. Although the coefficients of individualistic culture and fatalist culture were not significant, the coefficients were negative as we expected.

Hypothesis 3a expected that risk perception plays a mediating role for the relationship between culture and pro-environmental behavior. First, Model 1 shows that different cultural worldviews have different impacts on perception. While fatalistic culture has a significantly negative correlation with environmental risk perception, the other three cultures are positively correlated with environmental risk perception. In addition, the results in Model 2 suggest a significant positive correlation between risk perception and pro-environmental behavior, which is consistent with existing studies [8]. It supports Hypothesis 3b that the increase in environmental risk perception will increase individuals’ pro-environmental behavior. Finally, culture and environmental risk perception were included in the Model 4 and the results suggested that environmental risk perception is positively correlated with pro-environmental behavior at a significant level. However, the correlations between all cultural types and pro-environmental behaviors were significantly reduced when compared with Model 3. It suggests that that risk perception plays a mediating role for the relationship between culture and pro-environment behavior.

**Figure 6:**
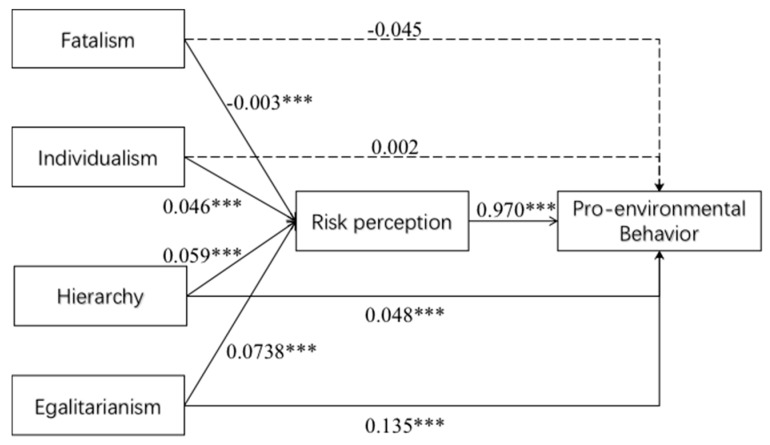
Path model explains the mediating effect of risk perception on the relationship between risk culture and pro-environmental behavior,*** denotes significance level of 1%

Path analysis is displayed in Figure 6. Hierarchy and egalitarianism are not only directly related to pro-environmental behavior, but also may increase pro-environmental behavior by increased environmental risk perception, showing that risk perception plays partial mediating role in this process (Figure 6).

In addition, the effects of control variables, including knowledge level on pro-environmental behavior are summarized in Table 6. Knowledge has a positive influence at a significant level. Young people in higher education are more likely to have a higher level of environmental risk perception and pro-environmental behavior. Also, young people who are majoring in environment related areas are more likely to be engaged in environmental protection. Therefore, we suggest that the increase of knowledge level of young people on environmental issues will facilitate the development of pro-environmental behavior. The gender variable is significantly negative, suggesting that women are more likely to participate in pro-environmental behavior than men, which is consistent with previous research findings [63,64].

### 4.3. Robust Check

In order to ensure the robustness of the research findings, this study used four dominant culture types to replace the cultural indices for regression analysis, and the dominant culture was the cultural type for which the cultural index was reported as highest. Therefore, dominant cultures are measured by categorical variables. The results are shown in Table 7, which is very similar to the results in Section 4.2. In particular, in model 8, the coefficient of risk perception is still significantly positive, and risk perception works as a mediator variable, explaining the positive correlation between egalitarian culture and pro-environmental behavior.

## 5. Brief Conclusion and Future Research

There are some limitations in this study. First of all, this research uses attitudinal items in an existing survey that approximate worldviews to measure culture, which decreases the construct validity of measurement. Future research should try to use the survey items directly measuring relations or cultural statement. However, some of consistent results with existing research on China [21,22] provide evidence that our measurements have predictive validity. Considering the condition that China does not have a large-scale survey using cultural measurements developed in the United States, our paper suggests that future research seeking for measuring cultures based on existing survey should select survey items regarding role of authority and government and preference for self-interest over other values. Second, there are some other important factors, such as age, were not tested. We have used grade instead of age because we believe that grade should be related with age in Chinese context. However, we also suspect that age and grade are not the same thing. Therefore, future research should include this demographic factor. Finally, future research should ask whether the influence of the cultural biases and risk perception on pro-environmental behaviors vary with different environmental policy areas such as energy, water, and wastes management.

To explain the attitude-action lag between risk perception and pro-environment behavior, prior research focused on the rational choice model or benefits and costs analysis. Unlike other studies, our paper used cultural biases to fill the gap of the previous research. Our research suggests that people think and act in such a way that cultural biases function as an orienting mechanism that helps people navigate a world full of uncertainties and risks [32]. In the case of young people’s pro-environmental behavior, culture constraints individuals’ core values and behavioral preferences in the face of current environmental issues [65,66,67].

Additionally, the results show that Chinese youth are more likely to be affiliated with hierarchical and egalitarian culture. Both of these two cultures belong to the high-group culture type in the grid-group cultural type quadrant. High group cultures emphasize a close connection between individuals and through collective identity, i.e., they foster a high level of collective thinking. This could be explained by China’s national sociocultural conditions. Both egalitarian and hierarchical cultures have a positive effect on the pro-environmental behavior among young people. However, contrary to our expectation, we found no influence of individualism on pro-environmental behaviors. We suggest one possible explanation is that individualists act on self-interests which is very context dependent. Compared with other cultures, they are more willing to act when their personal interests exceed social interests or their private costs are less than social costs. For example, people living in poverty could be less likely to act in protecting environment while people having better income could be more likely to support environmental protection. By contrast, the effect of self-interest might not influence egalitarians’ and hierarchists’ choices as much as individualism. In addition, there are many other factors, such as media coverage on environmental protection, family influences and pressures, religious and spiritual ideals, identities etc., that may influence environmental behaviors and mediate environmental value. Future research should test how other different factors influence environmental perception and behavior.

Our research results confirmed that risk perception plays a mediating role in the correlation between egalitarian culture and pro-environmental behavior. Although this has been studied in previous studies, we focused on Chinese young people by using a much larger sample size. Moreover, we found that environmental risk perception is significantly related to pro-environmental behavior. Therefore, raising the perception of environmental risks among young people can help young people develop pro-environmental behaviors.

Among control variables, the knowledge level of young people is also significantly related to pro-environmental behaviors. Therefore, we suggest that providing better education and improving environmental knowledge among youth may help them to understand environmental risks more comprehensively and objectively, and thus enhancing their pro-environmental behavior.

For the policy makers, we suggest that, individuals will understand and accept policy recommendations only when they recognize that policies are compatible with their cultural beliefs and in line with their cultural rationality. Therefore, to reach a consensus on environmental policies, the government should consider the varieties of the public’s environmental risk perceptions and their environmental awareness when formulating policies. The cultural attributes of environmental policies and governance should not be ignored.

## Figures and Tables

**Table 1:** Measuring Pro-environmental Behavior.

Key Themes	Description	Mean	Std.	Min	Max
Have you taken the initiative to learn about the ecological civilization?	5-point scale (1: never; 2: hardly; 3: occasionally; 4: often; 5: always)	2.943	0.738	1	5
Have you ever participated in the ecological civilization activities organized by the school?	4-point scale (1: have not participated and consider it unnecessary; 2: have not participated but consider it necessary; 3: have participated but thought it was not very useful; 4: have participated and thought it makes sense)	2.632	0.878	1	4
Will you make donations for eco-public activities?	4-point scale (1: never considered doing this; 2: depending on economic condition; 3: if there is such an activity around, I am very happy to participate; 4: already done this)	2.841	0.712	1	4
Have you ever promoted the knowledge of ecological civilization to the people around you?	5-points scale (1: never; 2: hardly; 3: occasionally; 4: often; 5: always)	3.044	0.782	1	5
Do you turn off the lights or air conditioners when you leave your bedroom or empty classroom?	5-points scale (1: never; 2: hardly; 3: occasionally; 4: often; 5: always)	4.549	0.687	1	5
Do you pay attention to the classification mark of the trash cans when you throw away garbage?	5-points scale (1: never; 2: hardly; 3: occasionally; 4: often; 5: always)	3.928	0.941	1	5
Do you pay attention to energy saving and environmental protection signs when buying goods?	5-points scale (1: never; 2: hardly; 3: occasionally; 4: often; 5: always)	3.056	1.014	1	5

**Table 2:** Measuring Culture.

Questions	Cultural Bias	Scores
What motivates you to protect the environment?	Fatalism: No motivation and irrelevant	Yes = 1, No = 0
	Individualism: Self interest	
	Hierarchy: Laws and rules	
	Egalitarianism: Respect for nature as human being	
Who should take the responsibility for environmental protection?	Fatalism: Others	Yes = 1, No = 0
	Individualism: Businesses enterprises	
	Hierarchy: Government	
	Egalitarianism: Every individual	
Attitude to commerce and industry?	Fatalism: Irrelevant	
	Individualism: Unrestricting	
	Hierarchy: Regulation	
	Egalitarianism: Anti-commerce	
Ranking of responsibility in environmental protection.	Fatalism: Others	Rank in 1st = 5 Rank in 2nd = 4 Rank in 3rd = 3 Rank in 4th = 2 Excluded by the respondents = 1
	Individualism: Business	
	Hierarchy: Government	
	Egalitarianism: Everyone	

**Table 3:** Summary of Variables.

Variables	Description	Mean	Std.	Min	Max	Obs.
**Dependent Variable**						
Pro-environmental behavior	Statistics on the degree and frequency of participation in environmental protection activities, such as participating in ecological civilization activities, turning off lights actively, etc.	22.995	3.398	7	28	8084
**Independent Variables**						
Environmental risk perception	Statistics on answers concerned about the current overall ecological environment in China	4.145	0.705	1	5	8084
Risk Culture	Fatalism	2.218	0.682	1	8	8084
	Individualism	3.627	1.383	1	8	8084
	Hierarchy	5.093	1.730	1	8	8084
	Egalitarianism	5.409	1.925	1	8	8084
**Control Variables**						
Knowledge						
Education	Completion of a master’s degree and above	0.0468	0.211	0	1	8084
Major Related	Majoring in ecological environment related area.	2.527	0.7456	1	4	8084
Demographic factors						
Gender	Male = 1, Female = 0	0.356	0.746	0	1	8084
Grade	Level of grade (1–4: from first year of university to fourth year undergraduate student; 5,6: from first year to second year graduate student; 7–9: from first year to third year Ph.D. student)	5.195	1.312	1	9	7980
School	Categorical variables indicating the school where students attended	49.093	42.269	1	153	8084
Province	Categorical variables indicating the provinces where students study at	13.801	7.652	1	30	8084

**Table 4:** Analysis of Variance.

Source	SS	Df	MS	F	Prob > F
Between groups	394.81626	2	197.40813	17.37	0.0000
Within groups	89,080.4379	7838	11.3652		
Bartlett’s test for equal variances: chi2(2) = 1.2849, Prob > chi2 = 0.526					

**Table 5:** Correlation matrix of main variables.

Variables	Behavior	Perception	Fatalism	Individuali-Sm	Hierarchy	Egalitaria-Nism
Behavior	1.00					
perception	0.232 ***	1.00				
Fatalism	−0.032 ***	−0.044 ***	1.00			
Individualism	−0.010	0.041 ***	−0.070 ***	1.00		
Hierarchy	−0.015	0.030 ***	−0.043 ***	0.075 ***	1.000	
Egalitarianism	0.087 ***	0.090 ***	−0.125 ***	−0.301 ***	−0.611***	1.000

Notes: *** denotes significance level 1%.

**Table 6:** Regression results (Risk Cultural Biases).

Variables	Perception	Behavior		
	Model 1	Model 2	Model 3	Model 4
Risk perception		1.008 ***		0.970 ***
		(0.058)		(0.057)
Fatalism	−0.003 ***		−0.048	−0.045
	(0.014)		(0.066)	(0.062)
Individualism	0.046 ***		−0.046	0.002
	(0.006)		(0.029)	(0.029)
Hierarchy	0.059 ***		0.106 ***	0.048 ***
	(0.006)		(0.029)	(0.029)
Egalitarianism	0.0738 ***		0.207 ***	0.135 ***
	(0.006)		(0.028)	(0.028)
Education	0.172 ***	0.555 ***	0.757 ***	0.590 ***
	(0.041)	(0.215)	(0.217)	(0.213)
Major Related	0.079 ***	0.923 ***	0.994 ***	0.917 ***
	(0.011)	(0.051)	(0.052)	(0.051)
Grade	−0.001	−0.013 ***	-0.022	0.023
	(0.017)	(0.078)	(0.080)	(0.078)
Gender	−0.032 ***	−0.134 ***	−0.154 ***	−0.123 ***
	(0.006)	(0.030)	(0.031)	(0.030)
School	−0.000	−0.000	−0.000	−0.000
	(0.000)	(0.000)	(0.000)	(0.000)
Province	−0.002	0.004	0.001	0.004
	(0.001)	(0.005)	(0.005)	(0.004)
Constant	3.282 ***	17.117 ***	19.507 ***	16.320 ***
	(0.095)	(0.316)	(0.427)	(0.469)
Observations	7980	7980	7980	7980
R²	0.037	0.095	0.063	0.099
F	23.40	99.25	44.7	66.31

Notes: robust standard errors in parentheses; *** denotes significance level of 1%.

**Table 7:** Regression results (Dominant Risk Culture).

Variables	Perception	Behavior		
	Model 5	Model 6	Model 7	Model 8
Risk perception		1.008 ***		0.998 ***
		(0.058)		(0.057)
Individualism	0.185 ***		0.305	0.121
	(0.053)		(0.275)	(0.268)
Hierarchy	0.192 ***		0.308	0.116
	(0.046)		(0.250)	(0.242)
Egalitarianism	0.222 ***		0.753 ***	0.531 ***
	(0.046)		(0.250)	(0.243)
Education	0.174 ***	0.555 ***	0.764 ***	0.590 ***
	(0.044)	(0.215)	(0.220)	(0.215)
Major Related	0.085 ***	0.923 ***	1.008 ***	0.923 ***
	(0.010)	(0.051)	(0.052)	(0.051)
Grade	−0.014	−0.013 ***	−0.033	0.010
	(0.016)	(0.078)	(0.080)	(0.078)
Gender	−0.032 ***	−0.134 ***	−0.158 ***	−0.126 ***
	(0.007)	(0.030)	(0.031)	(0.030)
School	−0.000	−0.000	−0.000	−0.000
	(0.000)	(0.000)	(0.000)	(0.000)
Province	−0.002	0.004	0.001	0.003
	(0.001)	(0.005)	(0.005)	(0.004)
Constant	3.941 ***	17.117 ***	20.750 ***	16.817 ***
	(0.064)	(0.316)	(0.331)	(0.404)
Observations	7980	7980	7980	7980
R²	0.015	0.095	0.056	0.098
F	13.79	99.25	47.50	73.41

Notes: robust standard errors in parentheses; *** denotes significance level of 1%.

## Appendix A

**Table A1:** Survey Items for Measuring Pro-environmental Behavior.

Survey Items in English	Survey Items in Chinese
Have you taken the initiative to learn about the ecological civilization? (1) Never (2) Hardly (3) Occasionally (4) Often (5) Always	你会主动了解有关生态文明的相关信息吗？ （1）从来不会 （2）几乎不会 （3）偶尔会（4）经常会 （5）总是会
Have you ever participated in the ecological civilization activities organized by the school? (1) I have not participated and consider it unnecessary. (2) I have not participated but consider it necessary. (3) I have participated but thought it was not very useful. (4) I have participated and thought it makes sense.	你是否参加过学校组织的生态文明活动？ （1）没有参加过但认为是必要的。（2）没有参加过但认为是必要的。（3）参加过但是认为没有太大用处。（4）参加过且认为意义重大。
Will you make donations for eco-public activities? (1) I never considered doing this. (2) It depends on economic condition. (3) If there is such an activity around, I am very happy to participate. (4) I have already done this.	你是否为一些生态公益活动进行爱心捐赠？ （1）从未考虑过这样做。（2）视经济情况决定。（3）如果身边有这样的活动，非常乐意参加。（4）已经在这样做了。
Have you ever promoted the knowledge of ecological civilization to the people around you? (1) Never (2) Hardly (3) Occasionally (4) Often (5) Always	你会主动向身边人宣传生态文明的知识吗？ （1）从来不会 （2）几乎不会 （3）偶尔会（4）经常会 （5）总是会
Do you turn off the lights or air conditioners when you leave your bedroom or empty classroom? (1) Never (2) Hardly (3) Occasionally (4) Often (5) Always	你在离开寝室、空教室时会主动关闭电灯或空调吗？ （1）从来不会 （2）几乎不会 （3）偶尔会（4）经常会 （5）总是会
Do you pay attention to the classification mark of the trash cans when you throw away garbage? (1) Never (2) Hardly (3) Occasionally (4) Often (5) Always	在路边丢弃垃圾时，你会主义垃圾桶的分类标志吗？ （1）从来不会 （2）几乎不会 （3）偶尔会（4）经常会 （5）总是会
Do you pay attention to energy saving and environmental protection signs when buying goods? (1) Never (2) Hardly (3) Occasionally (4) Often (5) Always	购买商品时，你会注意商品的节能环保标志吗？ （1）从来不会 （2）几乎不会 （3）偶尔会（4）经常会 （5）总是会

**Table A2:** Survey Items for Measuring Culture.

Survey Items in English	Survey Items in Chinese
What motivates you to protect the environment? (1) No motivation and irrelevant (2) Self interest (3) Laws and rules (4) Respect for nature as human being	当你做出保护生态的行为时出发点是？ （1）没有兴趣且与我无关（2）个人利益（3）法律规定（4）尊重自然
Who should take the responsibility for environmental protection? (1) Others (2) Business enterprises (3) Government (4) Every individual	你认为保护生态环境是谁的责任？ （1）其他（2）企业（3）政府（4）个人
What is your attitude towards industry and commerce? (1) Irrelevant (2) Unrestricting (3) Regulation (4) Anti-commerce	你对工商业的态度是什么？ （1）如我无关（2）减少限制（3）规则约束（4）反对商业
Ranking of responsibility in environmental protection. Government/Business enterprises/Every individual/Others	对于环境保护中的责任进行排序。政府／企业／个人／其他
